# The SCOOP12 peptide regulates defense response and root elongation in *Arabidopsis thaliana*

**DOI:** 10.1093/jxb/ery454

**Published:** 2019-02-04

**Authors:** Kay Gully, Sandra Pelletier, Marie-Charlotte Guillou, Marina Ferrand, Sophie Aligon, Igor Pokotylo, Adrien Perrin, Emilie Vergne, Mathilde Fagard, Eric Ruelland, Philippe Grappin, Etienne Bucher, Jean-Pierre Renou, Sébastien Aubourg

**Affiliations:** 1 IRHS (Institut de Recherche en Horticulture et Semences), UMR 1345, INRA, Agrocampus-Ouest, Université d’Angers, QuaSaV, Beaucouzé, France; 2 Institut Jean-Pierre Bourgin, INRA, AgroParisTech, CNRS, Université Paris-Saclay, Versailles, France; 3 iEES-Paris (Interaction Plantes-Environnement Institut d’Ecologie et des Sciences de l’Environnement de Paris), UMR CNRS 7618, Université Paris Est Créteil, 61 avenue du général de Gaulle, Créteil, France

**Keywords:** Arabidopsis, DAMP, defense signaling, oxidative stress, phytocytokines, root development, secreted peptide

## Abstract

Small secreted peptides are important players in plant development and stress response. Using a targeted *in silico* approach, we identified a family of 14 Arabidopsis genes encoding precursors of serine-rich endogenous peptides (PROSCOOP). Transcriptomic analyses revealed that one member of this family, *PROSCOOP12*, is involved in processes linked to biotic and oxidative stress as well as root growth. Plants defective in this gene were less susceptible to *Erwinia amylovora* infection and showed an enhanced root growth phenotype. In *PROSCOOP12* we identified a conserved motif potentially coding for a small secreted peptide. Exogenous application of synthetic SCOOP12 peptide induces various defense responses in Arabidopsis. Our findings show that SCOOP12 has numerous properties of phytocytokines, activates the phospholipid signaling pathway, regulates reactive oxygen species response, and is perceived in a BAK1 co-receptor-dependent manner.

## Teaser

A secreted peptide, a member of a Brassicaceae-specific gene family, acts on pathogen defense response and root development through the phospholipid pathway and ROS regulation.

## Introduction

In order to counter constant pathogen aggression, plants have developed sophisticated perception and defense systems. These plant responses are regulated by complex networks involving regulatory proteins and hormones, and are associated with massive changes in gene expression (Buscaill and Rivas, 2014). Among the involved players, it has been shown that small secreted peptides play an important role through their direct interaction with pathogens or through their function in development and cell–cell communication involving ligand–receptor interactions (Murphy *et al.*, 2012; Marmiroli and Maestri, 2014; Gust *et al.*, 2017). The secreted peptides derive from protein precursors having a shared N-terminal signal peptide which targets the protein to the secretory pathway. They can be categorized into two major classes: (i) the small post-translationally modified peptides (PTMPs) which are the targets of post-translational maturation and are produced through proteolytic processing; and (ii) the cysteine-rich peptides (CRPs) characterized by an even number of cysteine residues involved in intramolecular disulfide bonds (Tavormina *et al.*, 2015). Although they are mainly involved in plant growth and developmental processes, it has been shown that numerous genes encoding secreted peptides are also involved in plant defense mechanisms (Albert, 2013). For instance, the CRP class includes the antimicrobial peptides such as knottins and defensins, which interact with and disrupt the pathogen cell membrane (Goyal and Mattoo, 2014). Regarding PTMPs, families such as the phytosulfokines (PSKs), CLE/CLV3, IDA/IDL, or PSY are players in processes regulating a large panel of plant–pathogen interactions (Lee *et al.*, 2011; Shen and Diener, 2013; Vie *et al.*, 2015; Rodiuc *et al.*, 2016). Among secreted peptides, those showing immunity-inducing activity have been classified as damage/danger-associated molecular patterns (DAMPs) (Boller and Felix, 2009; Heil *et al.*, 2012). Through the action of lytic enzymes, a pathogen can penetrate the plant cell wall; the cell wall fragments released in this way into the apoplastic space can be perceived by neighboring cells, resulting in defense reactions. Oligalacturonides and cutin monomers are examples of non-peptidic DAMPs which are released upon fungal infection (Fauth *et al.*, 1998). Their perception by neighboring cells also elicits the immunity response (De Lorenzo *et al.*, 2011). The small peptide *At*Pep1 is a well-documented DAMP (Bartels and Boller, 2015). A first induction of *At*Pep1 and other peptides of this family by wounding or pathogen attack has a positive feedback on the expression of its own precursors as well as defense marker genes, that is thought to amplify defense signaling pathways (Huffaker and Ryan, 2007).

It is considered that only a small fraction of the gene space likely to encode signaling peptides has been described, and their diversity appears to be largely underestimated (Matsubayashi, 2014). Indeed, the Arabidopsis genome contains >1000 genes harboring secreted peptide features whose biological function is currently unknown (Lease and Walker, 2006, 2010). This lack of data can be explained by the fact that this type of gene has only recently been detected due to their small size and their low sequence conservation (Silverstein *et al.*, 2007). Furthermore, the frequent functional redundancy inside these gene families (Matsubayashi, 2014) renders mutant knock-out approaches less successful. The mining of previously published transcriptomes is an efficient way to explore this unknown gene space and decipher functions of new genes for which, without reference, the inference of function by similarity cannot be applied. Based on transcriptome meta-analysis and bioinformatics predictions in a ‘guilt by association’ approach, we identified a peptide family, of which at least one member is involved in plant immunity and root development. This work describes the identification of a gene family specific to the Brassicaceae genus encoding putative secreted peptides. The functional characterization of *PROSCOOP12*, one of its members in Arabidopsis, shows that this small gene could act as moderator in the response to different pathogen aggressions and in root development, presumably via controlling reactive oxygen species (ROS) detoxification. We illustrate that the small endogenous SCOOP12 peptide displays most properties of phytocytokines, processed and actively transported players in endogenous danger signals without cellular damage (Gust *et al.*, 2017).

## Materials and methods

### Plant material

Plant material used was wild-type *Arabidopsis thaliana* L. Heynh cultivar 6 Columbia (Col-0) as well as the cultivar Wassilewskija (Ws) and the mutants *proscoop12* (T-DNA line FLAG_394H10 in the Ws background; primers used for genotyping are detailed in Supplementary Table S1 at *JXB* online), *bak1-4* (T-DNA line SALK_116202), *fls2* (Gómez-Gómez and Boller, 2000), and *pepr1/pepr2* described by Flury *et al.* (2013). The *proscoop12* mutant in the Col-0 background was created using the CRISPR/Cas9 (clustered regularly interspaced short palindromic repeats/CRISPR-associated protein 9) approach . We searched *proscoop12* gene-specific single guide RNA (sgRNA) and potential off-target sites in the Arabidopsis Col-0 genome using the Crispor Tefor program (http://crispor.tefor.net). The 20 base long sgRNA with the sequence AAGAACTTGACCCATTTTTG was used. Soil-grown plants used for *Erwinia amylovora* and *Alternaria brassicicola* inoculations as well as all *in vitro* plants [on Murashige and Skoog (MS) medium] were grown under short-day conditions (photoperiod of 8 h light at 22 °C/16 h dark at 21 °C, with 70% relative humidity). Plants used for all other assays were grown under long-day conditions (photoperiod of 16 h light at 22 °C/8 h dark at 21 °C, with 60% relative humidity). *Brassica napus* (Darmor-bzh) and *Solanum lycopersicum* (Sweet Baby) were grown under short-day conditions.

### Plant inoculation with *E. amylovora*

Ws, Col-0, and the *proscoop12* mutant in both genotypes were grown for 5 weeks on soil. Four leaves of 20 plants were infiltrated with bacterial suspensions of the wild-type strain of *E. amylovora* CFBP1430 at a concentration of 10^7^ colony-forming units (cfu ml^–1^) in sterile water or were mock treated using a needleless syringe. Symptom severity was scaled as described in Degrave *et al.* (2008). For symptom rating (for Ws and *proscoop12*-Ws), at least 12 rosette leaves were used per condition in two biological replicates. Maximal symptoms appeared at 24 h or 48 h post-inoculation (hpi) depending on biological replicates. Therefore, representative experiments are presented at either 24 hpi or 48 hpi. For bacterial counting (for Col-0 and *proscoop12*-Col-0), samples were taken 3 d post-infection using a cork borer (d=5 mm) to cut one leaf disc per infected leaf. Leaf discs were ground in sterile water, diluted, and plated as droplets of 10 µl on LB plates. Plates were incubated, and colonies were counted the next day. Bacteria of 32 leaves of the wild type and *proscoop12* were extracted and quantified.

### Seed contamination and leaf infection by *A. brassicicola*

Fifty surface-sterilized seeds per Petri dish of Ws and pro*scoop12* were immersed in a solution containing *A. brassicicola* (strain abra43) with 10^3^ conidia ml^–1^ for 1 h and dried under sterile conditions. Leaves of Ws wild type and the *proscoop12* mutant were inoculated with 5 µl of an *A. brassicicola* solution, with a concentration of 10^3^ conidia ml^–1^. Symptoms were observed 6 d after infection. Necrotic areas were quantified using ImageJ. The experiments were repeated three times.

### Protection assay

Mature leaves of *A. thaliana* plants were infiltrated by needleless syringe infiltration with the indicated elicitor peptide or control solution and were kept under long-day growth conditions for 24 h. The *Pseudomonas syringae* pv *tomato* DC3000 strain was grown in overnight culture on YEB medium plates supplemented with appropriate antibiotics. Cells were harvested from the plate, re-suspended in sterile 10 mM MgCl, and diluted to an OD_600_ of 0.02. The bacterial solution was infiltrated into the pre-treated leaves with a needleless syringe. Plants were maintained at high humidity. Samples were taken using a cork borer (d=8 mm) to cut one leaf disc per infected leaf. Leaf discs were ground in 10 mM MgCl, diluted to the indicated concentration, and plated as droplets of 10 µl on YEB plates with the appropriate selection. Plates were incubated at 28 °C and colonies were counted 2 h after infection (0 dpi) as well as 1 d and 2 d post-infection. Eight plants were infected for each pre-treatment and sampling time point. The experiment was performed twice with similar results.

### Transcriptomic analysis

Microarray analysis was performed with the CATMA array v5 (Hilson *et al.*, 2004). Leaves were collected 24 h after inoculation from two independent biological replicates. Total RNA was extracted using the Qiagen RNeasy kit according to the supplier’s instructions. RNA integrity, cDNA synthesis, hybridization, and array scanning were performed as described in Lurin *et al.* (2004). cDNA from leaves inoculated with *E. amylovora* were hybridized against cDNA of leaves inoculated with water collected at the same time point. Statistical analysis was based on two dye swaps as described in Gagnot *et al.* (2008). To determine differentially expressed genes, a paired *t*-test on the log ratios was performed. Spots displaying extreme variance were excluded. The raw *P*-values were adjusted by the Bonferroni method, which controls the family wise-error rate. We considered as differentially expressed those genes with a Bonferroni *P*-value ≤0.05 Gagnot *et al.* (2008).

### Determination of gene expression by qPCR

Detached leaves of 3-week-old plants were collected and floated for 2 h in elicitor or control solution. After the treatment, material was frozen and ground in liquid nitrogen. RNA from 100 mg of tissue was extracted using the NucleoSpin RNA plant extraction kit (Macherey-Nagel Hoerdt, France). The DNase treatment was performed according to the manufacturer’s recommendations. For PCR, cDNA was synthesized from 10 ng of total RNA extract with oligo(dT) primers using Moloney murine leukemia virus reverse transcriptase according to the manufacturer’s instructions (Promega). For quantitative real-time reverse transcription–PCR (qPCR) in a 96-well format, the Chromo4™ System (Bio Rad) was used. Expression was normalized to that of the gene *ACR12* (AT5G04740, because of its constant transcription profile upon elicitor treatments) using the qGene protocol (Muller *et al.*, 2002). All the gene-specific primers used are detailed in Table S1.

### Seedling growth inhibition assay

Seedlings were germinated on MS agar and grown for 5 d before transferring one seedling per well to 24-well plates containing 500 µl of MS medium or MS medium supplied with the indicated elicitor peptide to a final concentration of 1 µM (six replicates per elicitor peptide treatment). Photos were taken, and fresh weight and root length were measured after a further 8 d. The root length of *proscoop12* and wild-type plants was determined on vertical MS plates.

### Elicitor peptides

Peptides of flg22 (QRLSTGSRINSAKDDAAGLQIA), *A. thaliana* Plant Elicitor Peptide 1 (*At*Pep1) (ATKVKAKQRGKEKVSSGRPGQHN), SCOOP12 (PVRSSQSSQAGGR), scSCOOP12 (GRPRSASSGSVQQ), SCOOP12 S5/7A (PVRSAQASQAGGR), SCOOP12 S5A (PVRSAQSQAGGR), and SCOOP12 S7A (PVRSSQASQAGGR) were obtained from Eurogentec SA (Angers, France) and diluted in water to the final concentration used for the assays.

### Measurement of reactive oxygen species

For ROS assays, leaf discs of 3-week-old soil-grown plants were placed into each well of a white 96-well plate (Thermo Scientific, Waltham, MA, USA) in 0.1 ml of water and kept in the dark overnight. For elicitation and ROS detection, horseradish peroxidase and luminol were added to a final concentration of 10 µg ml^–1^ and 100 µM, respectively. Luminescence was measured directly after addition of elicitor peptides in a FLUOstar OPTIMA plate reader (BMG LABTECH, Offenburg, Germany).

### Callose deposition

Leaf discs were vacuum infiltrated for 10 min with the indicated elicitor solution and kept floating in elicitor or control solution for 24 h. Leaf discs were then fixed and destained in 1:3 acetic acid/ethanol until leaf tissue was completely transparent. After washing the leaf discs in 150 mM K_2_HPO_4_ for 30 min, the plant material was stained for 2 h in 150 mM K_2_HPO_4_ and 0.01% aniline blue. Callose deposition was quantified with a Leica DM1000 microscope equipped with a Qimaging Micropublisher 3.3 RTV camera using a DAPI filter.

### Cell culture conditions

*Arabidopsis thaliana* cells were grown in a liquid MS-based (Duchefa-Kalys, France) growth medium (pH 5.6) with the addition of 2,4-dichlorophenylacetic acid (0.2 mg l^–1^), sucrose (30 g l^–1^), and KH_2_PO_4_ (0.2 g l^–1^). Cells were grown under continuous light (200 µE m^–2^ s^–1^) on a rotary shaker and subcultured weekly to fresh medium. For radiolabeling experiments, 7-day-old cell suspensions were used.

### Radioisotope labeling of phospholipids

Arabidopsis cells were aliquoted (7 ml) in individual flasks and kept for 3 h under mild rotation for equilibration. Radioisotope labeling was done by the addition of 53 MBq l^–1^ [^33^P]orthophosphate. Lipids were extracted according to Krinke *et al.* (2009). Lipids were separated by TLC using an acidic solvent system composed of chloroform:acetone:acetic acid:methanol:water (10:4:2:2:1, v/v/v/v) (Lepage, 1967) or in a solvent system composed of chloroform:methanol:ammonia:water (90:70:1:16, v/v/v) (Munnik *et al.*, 1994). Radiolabeled spots were quantified by autoradiography using a Storm phosphorimager (Amersham Biosciences, UK). Individual phospholipids were identified by co-migration with non-labeled standards visualized by primuline staining or by phosphate staining.

### Accession numbers

Transcriptome data are available at the Gene Expression Omnibus with the accession number GSE22683. The samples used (including biological repetitions) are: GSM562282, GSM562283, GSM562284, GSM562285, GSM562286, GSM562287, GSM562288, GSM562289, GSM562294, GSM562295, GSM562296, and GSM562297.

## Results

### Identification of the PROSCOOP gene family

**Fig. 1.**
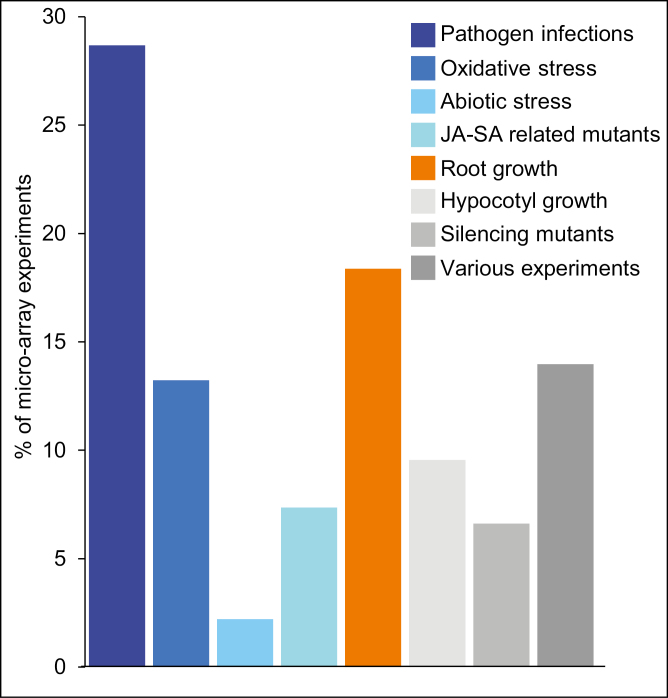
Synthesis of the results from the 136 experiments in which *AT5G44585* was significantly deregulated (Bonferroni *P*-value <5%) within the CATdb resource, then sorted into eight classes: pathogen infections, oxidative stress, abiotic stresses, JA–SA-related mutants, root growth, hypocotyl growth, silencing mutants, and various experiments. The whole set of results is detailed in Supplementary Table S2.

Meta-analysis of CATMA microarray data (Gagnot *et al.*, 2008) has previously highlighted several hundred non-annotated small protein-coding genes of unknown function in Arabidopsis (Aubourg *et al.*, 2007). Further, we investigated the whole CATMA resource available at this time in order to identify new genes induced by various stresses for further functional analyses. Among them, *AT5G44585* caught our attention because of its highly informative profile: this gene was differentially expressed in 136 experiments (21% of the whole set), being strongly induced in response to a large panel of biotic or oxidative stresses, *E. amylovora* infection being one of the top stresses. Biological contexts were extracted from each CATdb experiment (http://tools.ips2.u-psud.fr/CATdb) and classified into eight classes (Fig. 1; Supplementary Table S2). It is noteworthy that no less than 70% of the complete transcriptomic response of *AT5G44585* could be summarized with three keywords: pathogen response, oxidative stress, and root growth. Generally, we found this gene to be strongly up-regulated in most biotic and oxidative stress conditions, while it was down-regulated in conditions aiming at diminishing oxidative stress. Furthermore, *AT5G44585* exhibited a constitutive expression in roots in growth conditions but is down-regulated in numerous conditions affecting root elongation such as nitrogen starvation (Krapp *et al.*, 2011). This advocated for further exploration of this gene in oxidative stresses, root development, and in response to pathogen infections.

**Fig. 2.**
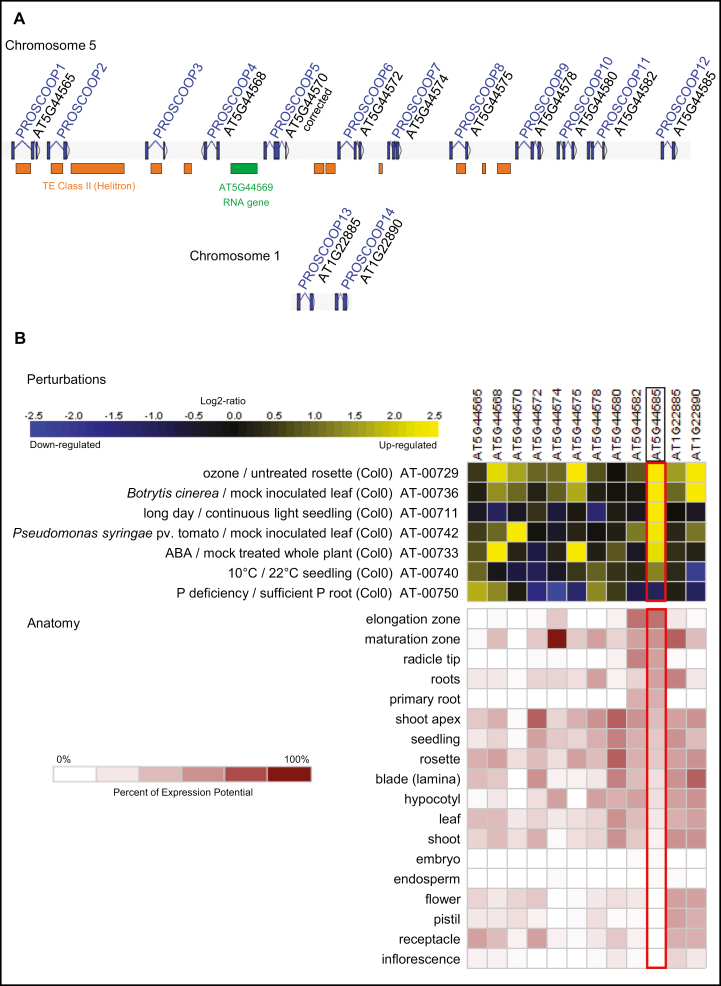
The *PROSCOOP* family. (A) Gene organization: coding exons and introns are represented by blue boxes and blue broken lines, respectively. Remains of transposable elements (Helitron type) are represented by orange boxes, and the green box indicates a putative non-coding RNA of unknown function. The TAIR gene names and corresponding *PROSCOOP* nomenclature are indicated. *PROSCOOP2* and *PROSCOOP3* are not annotated in the last TAIR version but are confirmed by the ESTs EG446167, EG448031, EG446890, and CB253842. (B) Transcription of the *PROSCOOP* family: significant (*P*-value <0.05) differential expression induced by specific perturbations (upper panel) and transcription level in different Arabidopsis organs (lower panel) are based on RNA-seq data obtained from the Genevestigator platform (Hruz *et al.*, 2008). The *PROSCOOP12* gene is indicated by a red frame.

The screening of the Arabidopsis genome revealed that *AT5G44585* belongs to a small family of 14 unknown homologous genes with similar intron–exon structure (two or three exons), encoding proteins ranging from 72 to 117 amino acids. Analysis of the N-terminal regions using the SIGNALP v4.1 (Nielsen, 2017) and the PREDOTAR v1.04 (Small *et al.*, 2004) software revealed a signal peptide targeting proteins to the endoplasmic reticulum to be present in all members of the family. DeepLoc v1.0 (Almagro *et al.*, 2017) predicts an extracellular localization for the 14 proteins, with scores ranging from 0.88 to 1. The 14 genes are organized in two tandemly arrayed clusters on chromosomes 1 and 5 (Fig. 2A). The largest 37 kb long gene cluster on chromosome 5 contains numerous vestiges of transposable elements (Helitron type) which could have impacted evolution of this family through local duplication events. Manual annotation revealed two additional yet non-annotated genes located between *AT5G44565* and *AT5G44568*. Both share significant similarities with the other tandemly arrayed homologs, and cognate ESTs validate their transcription. Our manual annotation also led to the correction of the structure of *AT5G44570* in which an overpredicted 3'-coding exon has been removed. The size of the proteins, the number and the organization of paralogs, the amino acid composition (notably the absence of cysteine), and the presence of a signal peptide are common features shared by the PTMP families previously published (Matsubayashi, 2014). Furthermore, as described below, we identified a short conserved motif in the C-terminal region of these proteins, candidate to be mature functional peptides after proteolytic processing. For these reasons, this newly identified family has been named PROSCOOP, for putative precursors of SCOOP peptide (Serine riCh endOgenOus Peptide). The genes are termed *PROSCOOP1*–*PROSCOOP14* (*AT5G44585* being *PROSCOOP12*) and the corresponding mature peptides are named SCOOP1–SCOOP14 (Fig. 2A).

Previously reported RNA sequencing (RNA-seq) approaches (Hruz *et al.*, 2008) allowed us to broaden our transcriptome analysis to the *PROSCOOP* family members that were missing on the microarrays (only four of them are present in the Affymetrix Ath1 chip). We could confirm the regulation of their transcription in several stress conditions and organs (Fig. 2B). These data show a large diversity of transcription profiles in this family, suggesting its involvement in different biological functions. Notably, *PROSCOOP12* shows a distinct transcription profile as it is among the minority of paralogs to be highly induced by aggression by different pathogens and expressed in the whole root system.

In order to assess the evolutionary conservation of the PROSCOOP family, an extensive BLASTP search for homologs in GenBank was carried out. We identified this family in several Brassicaceae genomes reaching from *Eutrema salsugineum* to *Camelina sativa*, and the number of identified homologs in these genomes ranged from 1 to 13. Outside the Brassicaceae genus, no similar proteins could be detected despite low stringency searches. The phylogenetic tree built from the multiple alignment of the 74 identified *PROSCOOP* homologs shows that gene duplications occurred before speciation of the eight different Brassicaceae species (Supplementary Fig. S1).

**Fig. 3.**
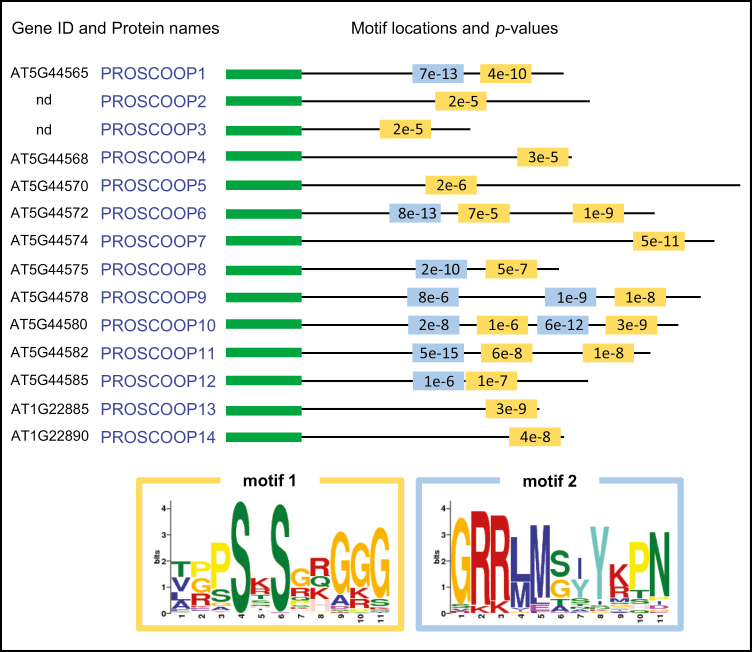
Conserved motifs identified in the PROSCOOP family proteins. The MEME v4.8.1 algorithm (parameters -nmotifs 3 -minw 6 -maxw 12) was run on the 74 homologous PROSCOOP proteins found in *Brassicaceae* genomes. *P*-values and motif locations are only shown for the 14 members from Arabidopsis. A third motif corresponding to the cleavage site of the signal peptides (green boxes) has also been highlighted by MEME and fits with SIGNALP v4.1 predictions.

In order to identify divergent yet still conserved smaller regions, the MEME algorithm (Bailey *et al.*, 2015) was used, excluding full-length alignments, on the 74 identified homologs. This sensitive approach allowed the identification of two significantly conserved 11 amino acid long motifs (Fig. 3). These motifs are good candidates for functional mature peptides (or a part of them) following the putative proteolytic processing of the corresponding precursor. Indeed, both motifs are proline, serine, arginine, and glycine rich, as in previously described PTMP families such as CLV3/CLE (Betsuyaku *et al.*, 2011), IDA (Vie *et al.*, 2015), PIP (Hou *et al.*, 2014), and CEP (Roberts *et al.*, 2013). Motif 1 is more ubiquitous than motif 2 since it was detected in 72 sites (e-value of 9.8e-213) compared with 39 sites (e-value of 3.4e-179) out of the 74 PROSCOOP homologs. Therefore, we have focused our downstream functional analysis on motif 1 (Fig. 3), named SCOOP hereafter.

### *PROSCOOP12* is co-expressed with genes involved in hormone signaling and defense

In order to make a first assessment of the potential biological relevance of *PROSCOOP12* and to predict its putative functional partners, we further mined previously published Arabidopsis transcriptome data (Gagnot *et al.*, 2008). Based on the assumption that genes with related biological functions are likely to be co-expressed (Schöner *et al.*, 2007), we used the results of the Gaussian mixture model-based clustering method from the GEM2Net resource (Maugis *et al.*, 2009; Zaag *et al.*, 2015). The *PROSCOOP12* gene was found to be co-expressed with 83 genes in a set of experimental samples comprising biotic stress triggered by necrotrophic bacteria and fungi. This cluster of 83 genes has been enriched by the integration of functional partners based on co-citations, protein–protein interactions, and common biological pathways using TAIR, the Arabidopsis interactome (Arabidopsis Interactome Mapping Consortium, 2011), and the STRING database (Szklarczyk *et al.*, 2017). This step resulted in a network of 117 genes (Supplementary Tables S3A, B) mainly focused on hormone crosstalk [in particular salicylic acid (SA)/jasmonic acid (JA) signaling], pattern-triggered immunity (PTI), brassinosteroid and phenylpropanoid pathways, and nitrogen metabolism (Suppplementary Fig. S2). Out of 117 genes, 53 are involved in response to stimulus (GO:0050896, fdr 1.31e-11); among them, 26 genes are classified in defense response (GO:0006952, fdr 5.72e-10) and 14 in transmembrane signaling receptor activity (GO:0004888, fdr 1.41e-09). Numerous key players in defense were found to be clustered with *PROSCOOP12*, such as the *NIMIN1*, *IOS1*, *NHL6*, *MLO12*, *FRK1*, *LECRKA4.1*, *CRK13*, and *HA2* genes and the *WRKY11*, -*14*, -*18*, -*22*, -*60*, and *-70* transcription factor genes. This relational network contains two other genes encoding PTMPs, namely *PROVIR10* and *PSK4*, and two PTMP receptor kinases, PSKR1 and PSY1R, that are involved in root development and modulation of SA/JA defense responses (Mosher *et al.*, 2013). *PROVIR10* has been found to correlate positively with disease triggered by necrotrophic pathogens (Dobón *et al.*, 2015) and *PSK4* encodes a phytosulfokine, one of the peptide growth factors involved in disease establishment (Rodiuc *et al.*, 2016). This approach led us to explore the role of *PROSCOOP12* and its SCOOP12 peptide regarding fungal and bacterial infections.

### PROSCOOP12 is involved in pathogen defense and root development

**Fig. 4.**
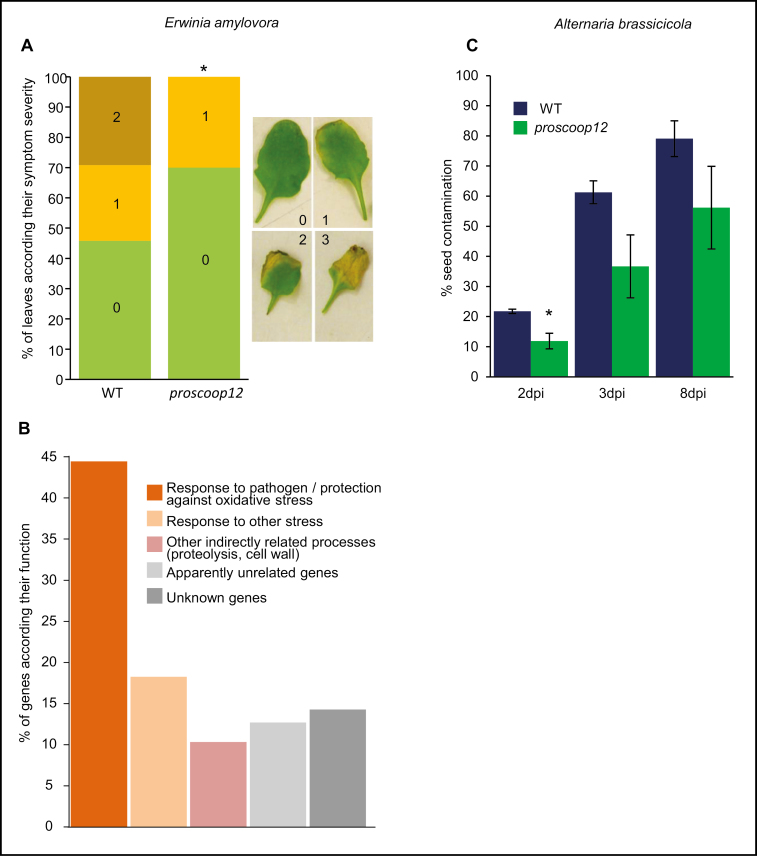
Mutant phenotype in response to *E. amylovora* and *A. brassicicola* infections. (A) Effect of *E. amylovora* infection on the *proscoop12* mutant. The symptom scale used (0–3) is illustrated on the right. The asterisk indicates a significant difference from symptom severity in wild-type leaves inoculated with *E. amylovora* (Mann–Witney test, α=0.05). (B) Distribution of the 126 genes up-regulated in *proscoop12* versus the wild type inoculated with *E. amylovora* according to their functional annotation. The complete results of this transcriptome approach are given in Supplementary Table S4. (C) Effect of *A. brassicicola* seed infection on *proscoop12* during germination 2, 3, and 8 d post-imbibition. Signiﬁcant differences according to Student’s *t*-test results: *P<0.05.

*PROSCOOP12* transcription was induced in the presence of different pathogens, *Erwinia amylovora* being one of the highest inducers (Fig. 1; Supplementary Table S2). Necrogenic pathogens are known to induce a response rather different from biotrophic pathogens in regards to ROS production (Venisse *et al.*, 2001). Therefore, in comparison with the responses of this gene to other oxidative stresses, we expected a high correlation. We hypothesized that infection with the necrogenic bacterium *E. amylovora* and the necrotrophic fungus *A. brassicicola* were suitable conditions to test a putative effect of the lack of function. This hypothesis was reinforced with the analysis of co-expressed putative partners, and its putative role as a secreted DAMP. Screening Arabidopsis mutant collections (Dèrozier *et al.*, 2011), we identified a T-DNA mutant *proscoop12* in the Ws background. Homozygous mutant plants did not transcribe *PROSCOOP12* (Supplemenatry Fig. S3). Compared with wild-type plants, *proscoop12* displayed a higher tolerance to *E. amylovora*-induced cell death as observed by a reduction of necrotic symptoms in leaves (Fig. 4A). This phenotype has only been observed in *wrky70* (Moreau *et al.*, 2012). Like *WRKY70*, *PROSCOOP12* acts as a negative regulator of defense against this bacterium. The transcription factor WRKY70 is known to positively regulate *WRKY60* and it is involved in the JA/SA crosstalk (Li *et al.*, 2004). Notably, these two genes have been found clustered with *PROSCOOP12* in our gene network analysis (Supplementary Fig. S2). We then performed a microarray transcriptomic comparison of *proscoop12* versus the wild type following bacterial inoculation. The results show that 3731 genes were differentially expressed in the wild type in response to *E. amylovora*, and 4125 in *proscoop12*. Despite the difference in symptom intensity, the vast majority of the bacteria-responsive genes did not display significant differences in both lines. Indeed, only 131 genes displayed a significantly different expression (Bonferroni *P*-value 5%) between wild-type- and *proscoop12*-infected plants (Supplementary Table S4): 126 up-regulated and 5 down-regulated genes, these latter corresponding only to hypothetical proteins or pseudogenes.

The 126 up-regulated genes that may contribute to the difference in symptoms between *proscoop12* and the wild type were challenged by functional annotation adding literature references to Gene Ontology (GO) terms to provide additional information (Supplementary Table S4; summarized in Fig. 4B). Indeed, 45% of them are connected to defense response (such as *HR4*, *SQP1*, *AED1*, *MKK2*, *HD2B*, and *NPR3*) and/or protection against oxidative stress (such as *ALDH24B*, *BiP2*, *APX1*, *ATOM1*, *APR1*, and *PER50*). Moreover, 18% were related to response to other stresses, mainly oxidative stress, and 10% could have indirect links with stress since they are involved in processes such as cell wall modifications or proteolysis. Only 13% could not be related to the phenotype, often because their function is currently unclear. Finally, the remaining 14% are unknown genes. The high percentage of genes directly related to protection against oxidative stress supports the hypothesis of a relationship between *PROSCOOP12* and the control of ROS production.

The response of *proscoop12* to a necrotrophic fungus infection was assessed using the Arabidopsis–*A. brassicicola* pathosystem (Pochon *et al.*, 2012). *Alternaria brassicicola* inoculation of rosette leaves produced similar symptoms in wild-type and *proscoop12* genotypes (Supplementary Fig. S4). Because seedling infection by *A. brassicicola* is mainly caused by seed transmission, we have also observed the fungal colonization during germination of infected seed lots under controlled conditions. Two days after sowing, *proscoop12* showed a significantly lower rate of germinating seeds prone to *A. brassicicola* infection compared with the wild type (Fig. 4C).

**Fig. 5.**
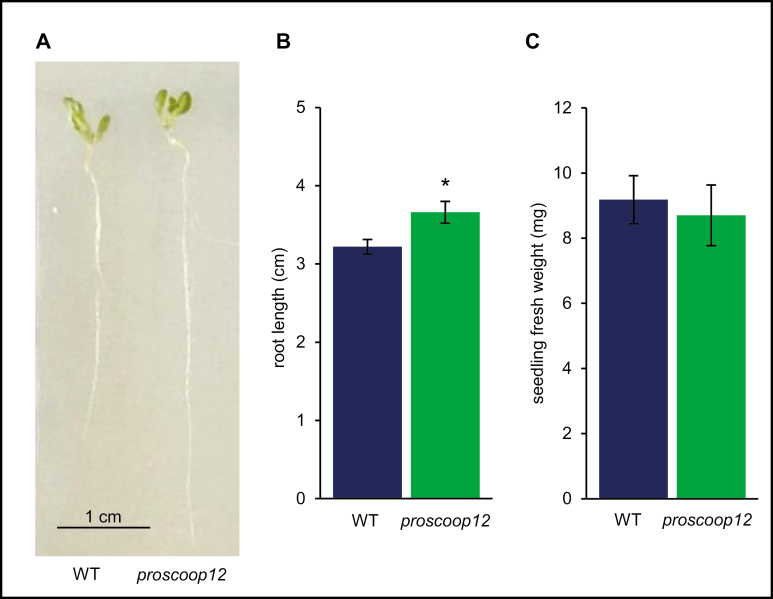
Phenotypic comparison between *proscoop12* and wild-type plants. (A, B) Root growth phenotypes determined after 10 d. Student’s *t*-test revealed that the different root length between the wild type and mutant is highly significant (*P<0.05). (C) Seedling fresh weight determined after 10 d. Bars show the combination of two biological repetitions (25 seedling each) and error bars show ±SE of the mean.

Because our transcriptome analysis suggested that *PROSCOOP12* may play a role in root development (Figs 1, 2B), we compared the root lengths of wild-type and *proscoop12* plants. Indeed, *proscoop12* plants developed significantly longer roots than control plants (Fig. 5A, B). No significant difference was observed between the wild type and *proscoop12* regarding the seedling fresh weight (Fig. 5C).

A second *proscoop12* line was obtained in the Col-0 background using a CRISPR/Cas9 approach. The frameshift obtained in the first exon disrupts the coding frame 10 amino acids after the editing event, upstream of the conserved motif. The phenotypes previously observed with the Ws *proscoop12* mutant were confirmed in this Col-0 mutant line (Suplementary Fig. S5).

### The SCOOP12 peptide has the main features of DAMPs

The structural features of the PROSCOOP12 protein suggested that it should be classified as a secreted PTMP. At the functional level, its transcriptional behavior suggested that it may play a role as a DAMP. Indeed, the induction of *PROSCOOP12* expression by a large panel of biotic stresses and the root phenotypes identified in the *proscoop12* mutant revealed some analogies with the *AtPROPEP1* and *AtPROPEP2* genes which are the precursors of the *At*Pep1 and *At*Pep2 peptides, respectively, well-characterized DAMPs (Bartels and Boller, 2015). Likewise, both genes are also induced by biotic stress (Huffaker *et al.*, 2006), and the *At*Pep1 DAMP is involved in root development since the overexpression of *AtPROPEP1* and *AtPROPEP2* causes significantly longer roots (Huffaker *et al.*, 2006). Therefore, we wanted to test if *PROSCOOP12* encodes a peptide that may act as a DAMP by comparing it with *At*Pep1.

#### The SCOOP12 peptide induces immune responses in Arabidopsis

**Fig. 6.**
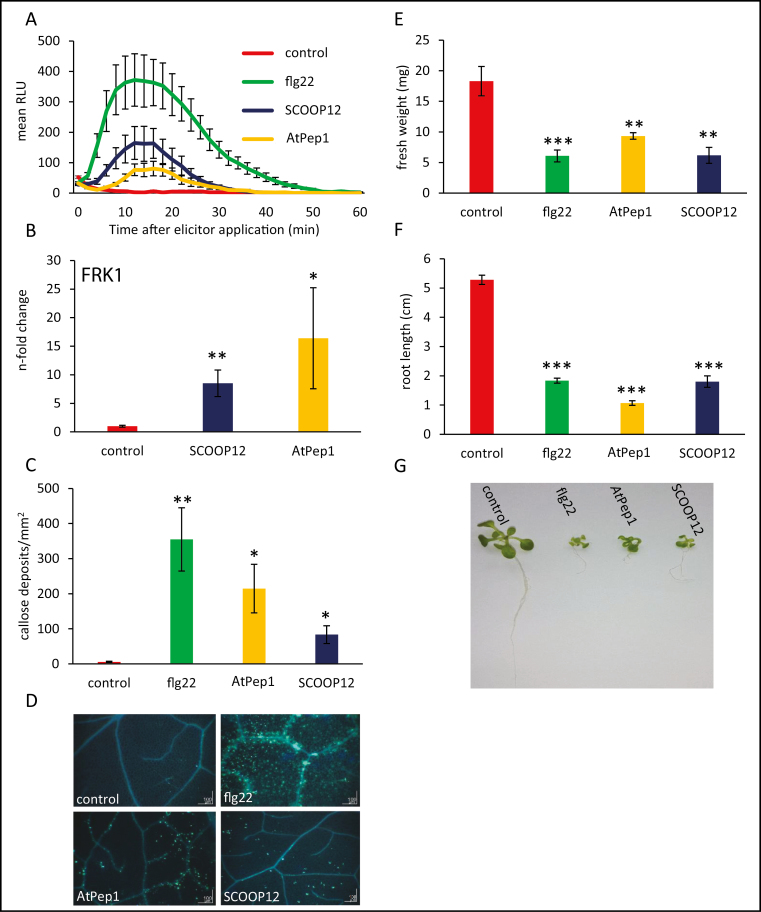
Defense responses induced by SCOOP12. (A) Production of reactive oxygen species (ROS), in RLU (relative light units), in wild-type Arabidopsis leaf discs (Col-0), treated with 1 µM of each peptide or without elicitor (control). Graphs display averages of 12 replicates. Error bars show the ±SE of the mean. (B) Induction of *FLG22-INDUCED RECEPTOR-LIKE KINASE1* (*FRK1*) gene transcription in soil-grown plants treated with 1 µM of the indicated peptide or without elicitor (control). Error bars show the ±SD of the mean based on three biological replicates. (C) Quantification of callose deposition. Error bars represent the ±SE of the mean of four replicates. (D) Localization of callose deposition by aniline blue staining. (E–G) Quantification of seedling growth inhibition. Five-day-old seedlings were transferred from solid MS medium to liquid medium supplied with the indicated elicitors (all applied at a final concentration of 1 µM) and grown for an additional 8 d before fresh weight and root length was quantified and pictures were taken. For all experiments, error bars show ±SE of the mean of six biological replicates. Signiﬁcant differences according to Student’s *t*-test results: *P<0.05; **P<0.01; ***P<0.001.

Based on the identification of the conserved motif 1 (Fig. 3), a putative mature peptide SCOOP12 was defined (PVRSSQSSQAGGR) from PROSCOOP12 and synthetized in order to explore its biological function. Despite the non-predictable post-translational modifications, we tested the exogenous application of the synthetic SCOOP12 peptide as previously described for CLE and RGF PTMP families (Matsuzaki *et al.*, 2010; Murphy *et al.*, 2012; Whitford *et al.*, 2012). Treatment of plants with SCOOP12 induced a wide range of long- and short-term immune responses (Fig. 6). One of the fastest defense responses is the production of ROS (Torres *et al.*, 2006). We show here that SCOOP12 induced a more intensive ROS burst compared with *At*Pep1 but weaker than flg22 (Fig. 6A). Next, we wanted to study the effect of SCOOP12 on genes closely linked to early defense mechanisms. *FRK1* has previously been shown to be induced by pathogens, elicitors, SA (Asai *et al.*, 2002; Boudsocq *et al.*, 2010), and *At*Pep1 (Flury *et al.*, 2013). Furthermore, our co-expression network approach identified co-expression of *PROSCOOP12* with *FRK1* (Supplementary Fig. S2). Therefore, we measured the *FRK1* expression level in detached leaves floating for 2 h in solutions supplemented by SCOOP12 or *At*Pep1. Compared with controls, *At*Pep1 and SCOOP12 treatments resulted in a 15-fold and 8.5-fold increase in *FRK1* expression, respectively (Fig. 6B). The deposition of callose is also known to be triggered by DAMPs (Luna *et al.*, 2011). Callose staining after 24 h of treatment with the elicitor peptides showed that SCOOP12 induces callose deposition, yet at a weaker level compared with flg22 or *At*Pep1 (Fig. 6C, D). One of the long-lasting defense responses is an inhibition of growth caused by the elicitor (Krol *et al.*, 2010). Our results indicate that perception of SCOOP12 also leads to an arrest of growth. The effect is comparable with the flg22 and the *At*Pep1 DAMP (Fig. 6E–G).

**Fig. 7.**
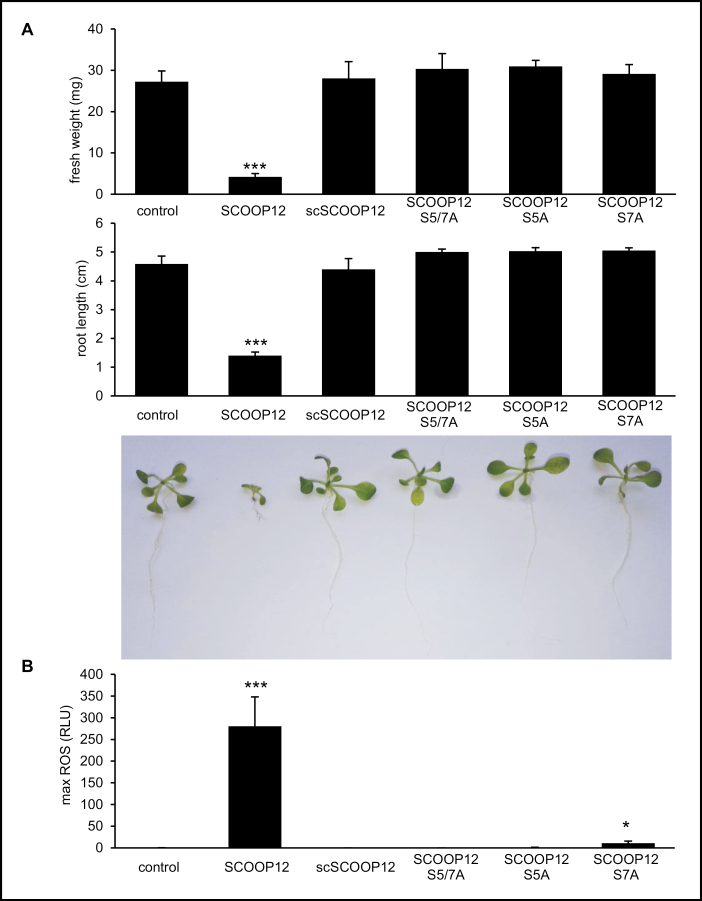
SCOOP12 activity depends on the correct amino acid order and two highly conserved serine residues. Assays were carried out with scrambled peptide (scSCOOP12) and alanine replacements of conserved serine residues in position 5 and 7 of SCOOP12 (PVRS***S***Q***S***SQAGGR) (SCOOP12 S5/7A; SCOOP12 S5A; and SCOOP12 S7A). (A) Quantification of seedling growth inhibition with the indicated elicitors. Bars of quantified fresh weight and root length represent the mean of six replicates. (B) Production of reactive oxygen species (ROS), in RLU (relative light units), in wild-type Arabidopsis leaf discs (Col-0), treated with 1 µM of each peptide or without elicitor (control). Graphs display averages of 12 replicates. Error bars show ±SE of the mean. Signiﬁcant differences according to Student’s *t*-test results: *P<0.05; ***P<0.001.

In order to demonstrate the specificity of the SCOOP12 sequence, we synthesized a peptide based on a randomized version of the same amino acids and tested plant responses to this scrambled SCOOP12 (scSCOOP12). Furthermore, we synthesized peptides with double alanine replacements (SCOOP12 S5/7A) and single replacements (SCOOP12 S5A and SCOOP12 S7A) to test the importance of the two highly conserved serine residues on positions 5 and 7 of SCOOP12 (Fig. 3) for its activity. Plants treated with scSCOOP12 as well as with the modified peptides did not show seedling growth inhibition. Total seedling fresh weight as well as root length were not different from those of control plants (Fig. 7A). Finally, treatments with scSCOOP12, SCOOP12 S5/7A, and SCOOP12 S5A did not induce a ROS burst, and only SCOOP12 S7A resulted in a low, but still significant ROS burst (Fig. 7B). These results highlight the importance of the amino acid order and the highly conserved serine residues for the perception of SCOOP12 by the plant.

Next, we wanted to test the conservation of plant responses to SCOOP12. For that purpose, plants were selected in which we identified *PROSCOOP* homologs (*B. napus*, Supplementary Fig. S1) and plants that do not contain this gene family (*Nicotiana benthamiana* and *S. lycopersicum*). We measured ROS production following application of SCOOP12 in these plants and included flg22 as a positive control. We detected a ROS burst caused by flg22 in all four plant species. On the other hand, SCOOP12 only resulted in a ROS burst in *A. thaliana* and, at a lower, yet still significant, level in *B. napus* (Supplementary Fig. S6). SCOOP12 seems to be similar enough to its closest *B. napus* homolog (BNCDY22858 with the motif FAGPSSSGHGGGR) to trigger a ROS burst. Therefore, only the two plant species containing homologs of the *PROSCOOP* gene family members showed a response to SCOOP12 treatments.

#### Pre-treatment with the SCOOP12 peptide protects Arabidopsis against *Pseudomonas* infection

**Fig. 8.**
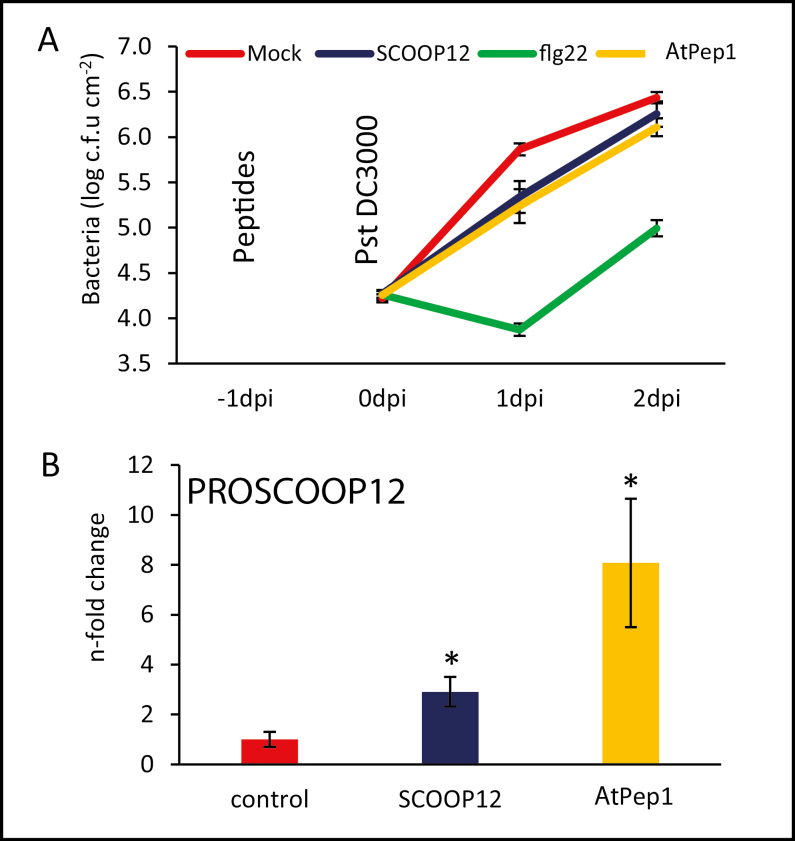
SCOOP12 application protects against *Pseudomonas* infection. Arabidopsis wild-type (Col-0) plants were pre-treated for 24 h by leaf infiltration with 1 µM of the indicated elicitor or without peptide. Subsequently, leaves were infected with 105 cfu ml^–1^*Pst*. DC3000, and bacterial growth was assessed 1 d and 2 d after infection. The plot represents the mean of eight replicates and error bars show the ±SE of the mean. Except between *At*Pep1 and SCOOP12, all differences are statistically significant at 1 d and 2 d after infection (*P*<0.05).

It has previously been shown that priming of plants with the flg22 elicitor as well as with oligogalacturonides could result in enhanced tolerance against subsequent bacterial infections. For instance, plants pre-treated with these elicitors showed significantly reduced lesion size following an infection with *Botrytis cinerea* (Raacke *et al.*, 2006; Ferrari *et al.*, 2007). Using a similar assay, we found that plants pre-treated with flg22 as well as with SCOOP12 and *At*Pep1 were less susceptible to *P. syringae* pv. *tomato* DC3000 infection (Fig. 8). The effect of the two endogenous peptides SCOOP12 and *At*Pep1 was weaker than that of flg22, which is consistent with the fact that flg22 induced a stronger defense response compared with SCOOP12 (Fig. 6A, C).

#### SCOOP12 and *At*Pep1 induce the expression of several *PROSCOOP* genes

It has previously been shown that small endogenous peptides can induce the expression of their own precursors, resulting in a positive feedback loop. For instance, expression of several *PROPEP* genes can be induced by different *At*Pep peptides (Huffaker and Ryan, 2007). This led us to investigate the change in the steady-state transcript level of all 14 *PROSCOOP* family members after SCOOP12 exposure. Moreover, we decided to add *At*Pep1 in our assay for comparison since it is also known to induce the transcription of another peptide precursor, *prePIP1* (Hou *et al.*, 2014). The results show that *PROSCOOP 2*, *7*, *8*, *12*, and *13* are up-regulated by the *At*Pep1 treatment (Supplementary Fig. S7). Most importantly, the direct precursor *PROSCOOP12* is up-regulated by SCOOP12 in comparison with the control treatment (Supplementary Fig. S7L). Therefore, there is a positive feedback loop linking SCOOP12 to its precursor *PROSCOOP12* but also of other members of the *PROSCOOP* family such as *PROSCOOP7*. However, SCOOP12 did not induce the expression of *PROPEP1* (Supplementary Fig. S7O). These results suggest that there is a feedback loop of SCOOP12 to its precursor and to *PROSCOOP7*, and that *At*Pep1 is capable of inducing five members of the *PROSCOOP* family.

#### The BAK1 co-receptor is involved in SCOOP12 perception

**Fig. 9.**
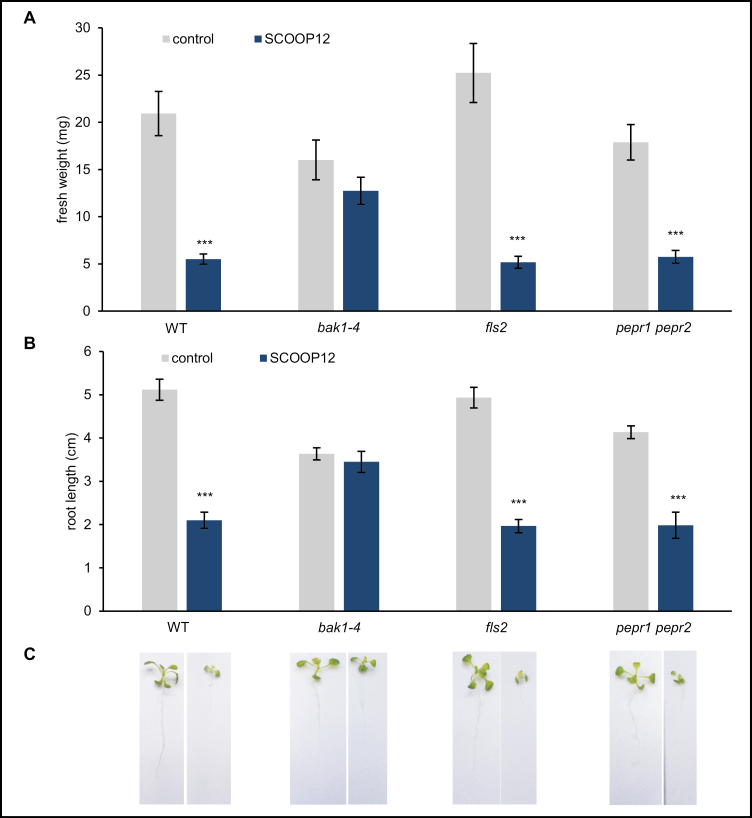
Seedling growth inhibition assay on selected receptor mutant backgrounds. (A) Fresh weight. (B) Root length. (C) Pictures of seedlings after 8 d of treatment. Neither fresh weight nor root length was affected by SCOOP12 treatment of *bak1-4* plants. The *fls2* and *pepr1*/*pepr2* receptor mutants were not affected in their perception of SCOOP12. Plants were grown for 8 d in the presence of 1 µM SCOOP12 or control solution. Bars of quantified fresh weight and root length represent the mean of six replicates. Error bars show ±SE of the mean. Signiﬁcant differences according to Student’s *t*-test results ***P<0.001.

A well-characterized co-receptor of several receptors of small peptides is BRI1-associated kinase1 (BAK1). Interaction of BAK1 with receptor-like kinases that act as elicitor receptors was proposed to be due to conformational changes occurring after ligand binding which results in the formation of the receptor complex (Chinchilla *et al.*, 2009; Liu *et al.*, 2017). To test if BAK1 is involved in the perception of SCOOP12, a seedling growth inhibition assay was performed on *bak1-4* plants. Compared with wild-type controls, *bak1-4* plants did not display any significant growth inhibition upon SCOOP12 treatment (Fig. 9). The same approach was carried out on *fls2* (the flg22 receptor) and *pepr1/pepr2* plants. In contrast to BAK1, our results suggest that these receptors are not involved in the perception of SCOOP12 (Fig. 9).

### SCOOP12 rapidly activates phospholipid signaling pathways in Arabidopsis cell suspensions

**Fig. 10.**
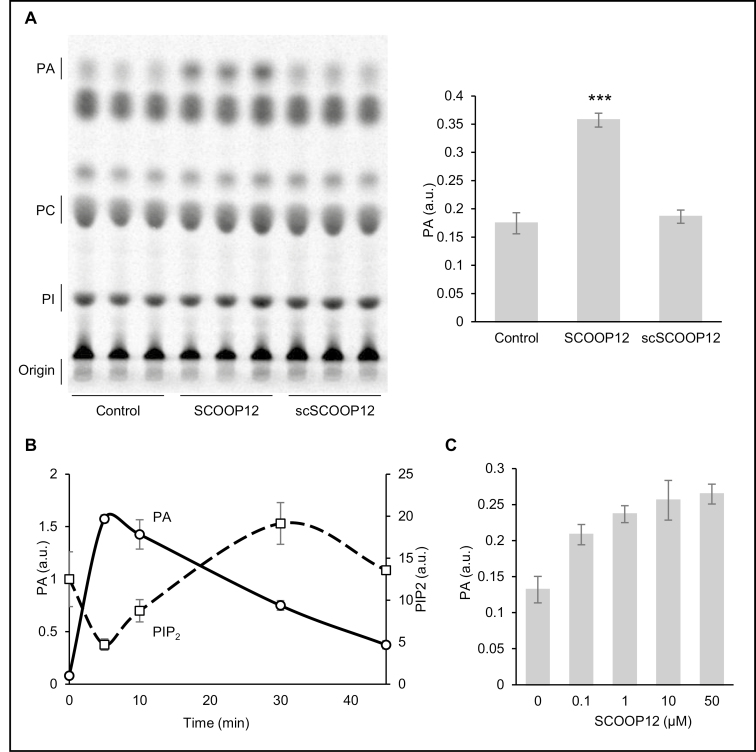
Rapid activation of PA production in Arabidopsis cell suspensions following treatment with SCOOP12. (A) Separation of ^33^P-labeled lipids using TLC with contrasting effects of SCOOP12 (10 µM) and scrambled scSCOOP12 (10 µM) on the level of PA accumulation visible after 5 min of treatment. Signiﬁcant differences according to Student’s *t*-test results: ***P<0.001. (B) Time scale of the SCOOP12 (1 µM) influence on PA and PIP_2_ accumulation in Arabidopsis cell suspensions. (C) Dose scale of the influence of SCOOP12 on PA and PIP_2_ accumulation in Arabidopsis cell suspensions after 5 min of treatment. All experiments were performed with at least three biological replicates. Error bars show ±SD of the mean. PA, phosphatidic acid; PIP_2_, phosphatidylinositol 4,5-bisphosphate; PI, phosphatidylinositol; PC, phosphatidylcholine; a.u., arbitrary units.

Lipid signaling pathways act as multifunctional regulatory mechanisms in plants. They incorporate several groups of inducible enzymes that convert membrane phospholipids into signaling molecules. Phosphatidic acid (PA) is a well-known biologically active lipid that is produced in response to numerous hormonal and stress signals including, notably, flg22 (van der Luit *et al.*, 2000). We demonstrate that application of SCOOP12 induces an accumulation of PA in Arabidopsis cell suspensions (Fig. 10A). This effect is observed as early as 5 min following SCOOP12 application at a low concentration of 100 nM (Fig. 10B, C). The scSCOOP12 had no effect on PA accumulation. Two modes of PA accumulation are known: phospholipase D (PLD)-dependent via direct hydrolysis of membrane phospholipids and diacylglycerol kinase (DGK)-dependent via phosphorylation of diacylglycerol (DAG). In our experiment, a labeling protocol that favors visualization of DGK-derived PA was used (Arisz and Munnik, 2013). Phosphatidylinositol 4,5-bisphosphate (PIP_2_) is a substrate to phosphatidylinositol-specific phospholipase C (PI-PLC) that produces DAG. We have also observed that the level of PIP_2_ is transiently reduced following SCOOP12 treatment (Fig. 10B). These results suggest that SCOOP12 initiates a signaling cascade implicating PI-PLC (causing the depletion of PIP_2_) and subsequent production of PA via phosphorylation of DAG by DGK.

## Discussion

**Fig. 11.**
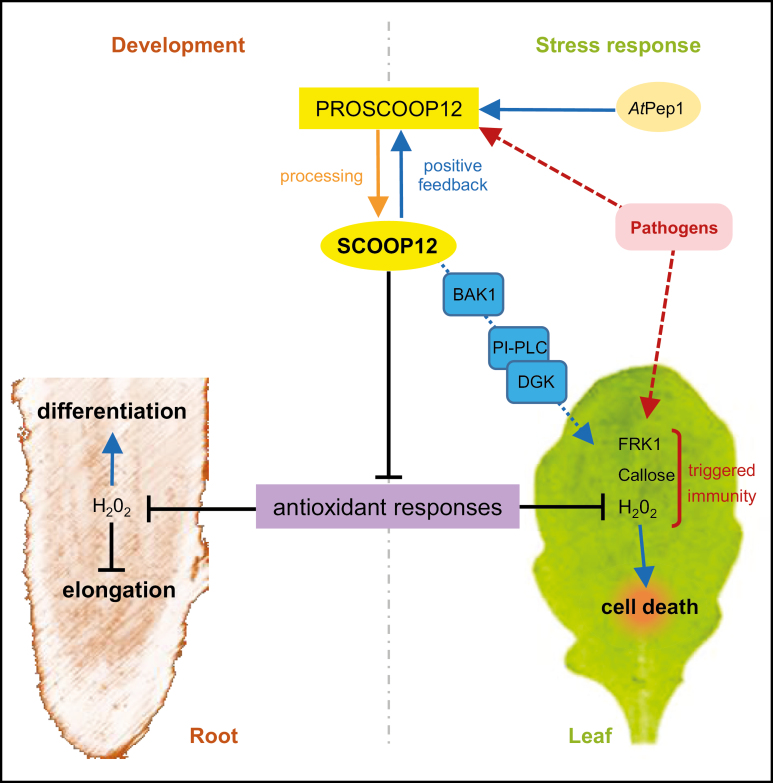
Putative model explaining the SCOOP12 functions in root development and biotic stress response through the inhibition of protection against oxidative stress. The red dotted arrows represent the action of the pathogens; the induction and the repression effects are represented by blue and black lines, respectively. PI-PLC, phosphatidylinositol-specific phospholipase C; DGK, diacylglycerol kinase.

Considered jointly, our transcriptome, mutant phenotyping, and peptide assay results allow us to propose a model explaining the roles of the SCOOP12 peptide in Arabidopsis (Fig. 11). The induction of numerous genes involved in the protection against oxidative stress such as peroxidases, glutathione transferase, and phenylpropanoid synthases in *proscoop12* in response to *E. amylovora* infection (Supplementary Table S4) might indicate that its lack of expression could result in a decrease in H_2_O_2_ levels. This could impair *E. amylovora* progression in leaves, which is known to induce H_2_O_2_ production in plants in order to promote cell death and invade plant tissues (Venisse *et al.*, 2001; Degrave *et al.*, 2008). In parallel, it is known that antioxidant responses in roots decrease the H_2_O_2_ level in the elongation zone, thereby contributing to root growth (Dunant *et al*., 2007; Tsukagoshi *et al.*, 2010). The constitutive expression of *PROSCOOP12* in roots (Fig. 2) could therefore contribute to higher levels of H_2_O_2_ and act as a moderator of root elongation under normal conditions. This is consistent with the greater root length observed in *proscoop12* (Fig. 5) and with the decrease of *PROSCOOP12* expression in roots in conditions leading to root lengthening such as nitrogen starvation (Supplementary Table S2).

In addition to its function in root elongation, we found *PROSCOOP12* to be involved in response to biotic stress in aerial parts where its transcription is strongly induced in the presence of pathogens (Figs 1, 2B). This induction triggers a ROS burst, putatively through the inhibition of the antioxidant responses, and then participates in the increase of H_2_O_2_ level in the infected tissues. This mechanism occurs when we apply the synthetic SCOOP12 peptide to seedlings, as illustrated by its induction of ROS burst, transcription of the *FRK1* defense gene, and callose deposition in leaf cells (Fig. 6). SCOOP12-induced PA production (Fig. 10) can be a part of a signaling cascade implicating several PA-binding proteins (Pokotylo *et al.*, 2018). PA binds NADPH oxidase isoforms D and F and stimulates NADPH oxidase activity in guard cell protoplasts (Zhang *et al.*, 2009). That is why PA production is likely to be upstream of ROS accumulation observed in response to SCOOP12. We have shown that the effects of SCOOP12 are BAK1 dependent (Fig. 9). It is known that the activity of BAK1 in receptor complexes is dependent on its phosphorylation state and is controlled by protein phosphatase 2A (PP2A) (Segonzac *et al.*, 2014). PA interacts with the scaffolding A1 subunit of PP2A, tethers it to membranes, and induces its activity (Gao *et al.*, 2013). This process was highlighted in connection with PIN1 dephosphorylation by PP2A in the auxin signaling cascade. However, similar reactions are to be expected for BAK1 dephosphorylation in PAMP/DAMP receptor complexes and indicate that they may act as an intrinsic part of the SCOOP12 regulatory cascade in plants.

The negative action of SCOOP12 on the antioxidant response is consistent with the reduction of symptoms observed in the *proscoop12* defective mutant in the presence of the necrogenic bacterium *E. amylovora* (Degrave *et al.*, 2008). In this case, the suppression of *PROSCOOP12* seems to enhance the protection against oxidative stress, thus hampering bacterial development in infected Arabidopsis leaves.

The comparison of the PROSCOOP family with other previously published genes encoding such secreted peptides highlights numerous shared features but also interesting specificities. At the structural level, the PROSCOOP proteins distinguish themselves by the absence of a highly conserved C-terminal region. Indeed, the motifs detected with the MEME tool are quite divergent compared with the other PTMP precursors (Matsubayashi, 2011). This divergence may explain the fact that no PROSCOOP homologs could be detected outside the *Brassicaceae* genomes. This restricted phylogenetic profile is opposite to the other described secreted peptides which are conserved in both monocots and eudicots. Furthermore, in contrast to the majority of the known PTMPs, the conserved motifs are not localized at the C-terminal extremity of their precursors, and their maturation could involve two steps of proteolytic processing or a trimming step (Matsubayashi, 2011). Out of the 14 Arabidopsis PROSCOOP proteins, three include two duplicated SCOOP motifs (Fig. 3), reminiscent of the previously described cases of the CEP and PIP families (Roberts *et al.*, 2013; Vie *et al.*, 2015) and also of the CLE18 protein in which each copy of the conserved CLE motifs has a specific function (Murphy *et al.*, 2012). The motif composition classifies SCOOP in the superfamily of ‘SGP-rich peptide’ among PIP, CLE, IDA, PEP, and CEP families (Hou *et al.*, 2014). At the functional level, the triggering of ROS burst, *FRK1* transcription, and callose deposition moves SCOOP12 close to the cytosolic *At*PEP and apoplastic PIP families (Huffaker *et al.*, 2006). Our results suggest a functional link between *At*Pep1 and SCOOP12 since both peptides induce the transcription of *PROSCOOP12* (Supplementary Fig. S7L). This collaboration between different peptide families has also been described with *At*PEP1 and PIP1 which act co-operatively to amplify triggered immunity. Furthermore, the signaling induced by *At*Pep1 (Schulze *et al.*, 2010), PIP1 (Hou *et al.*, 2014), and SCOOP12 (Fig. 9) is dependent on the BAK1 co-receptor. In addition to their role as amplifiers of the immune response, these peptides are involved in root development but via different mechanisms. The overexpression of the PIP1 precursor or its exogenous application inhibits Arabidopsis root growth as described for CEP (Roberts *et al.*, 2013) and SCOOP12 peptide (Fig. 6F). On the other hand, the constitutive overexpression of *PROPEP1* increases root development (Huffaker *et al.*, 2006) whereas *At*Pep1 treatment inhibits root growth (Poncini *et al.*, 2017). Acting as growth factors and in contrast to SCOOP12, the PTMPs PSK and PSY1 are involved in root elongation (Amano *et al.*, 2007; Matsuzaki *et al.*, 2010). These comparisons show that despite common structural and functional characteristics, the SCOOP family is different from previously described secreted peptides. The divergence observed in the C-terminal sequence of PROSCOOP proteins suggests a broad range of biological functions through a diversity of receptors which will be the targets of future studies.

In conclusion, SCOOP12 belongs to a new family of putatively secreted peptides specific to the Brassicaceae species. At the functional level, such secreted peptides are classified as phytocytokines (such as RALFs, systemin, and PIPs) which are secondary endogenous danger signals. Indeed, this classification (Gust *et al.*, 2017) distinguishes them from classical DAMPs (primary endogenous danger signals) which are passively released from injured tissue without​ biosynthesis and secretion processes. Nevertheless, the final processing of SCOOP12 is based on structural comparisons with analogous peptides and remains to be experimentally confirmed. Through its negative action on antioxidant responses and its positive effect on PA/ROS production (PLC pathway), SCOOP12 could play a role in the moderation of defense responses, as well as root elongation, to prevent unnecessary energy loss in a ‘trade-off’ fashion (Walters and Heil, 2007). The functions of such plant secreted peptides at the boundaries of development and stress signaling pathways open the way to future strategies that jointly consider product quality/quantity and new resistance traits.

## Supplementary data

Supplementary data are available at *JXB* online.

Fig. S1. Phylogenetic tree of *PROSCOOP* homologs.

Fig. S2. Relational annotation of genes co-expressed with *PROSCOOP12* and their functional partners.

Fig. S3. Confirmation of absence of transcription in the *proscoop12* T-DNA knock-out line by RT–PCR.

Fig. S4. Effect of *A. brassicicola* infection on *proscoop12* leaves.

Fig. S5. Confirmation of *proscoop12* mutant phenotype in a second genotype.

Fig. S6. ROS burst measurements on selected plant species treated with SCOOP12.

Fig. S7. Transcriptional response of the *PROSCOOP* gene family to SCOOP12 and *At*Pep1.

Table S1. Gene-specific primer sequences used for mutant genotyping and qPCR analysis of all the *PROSCOOP* genes.

Table S2. List of the 136 comparisons in which transcription of *AT5G44585* was deregulated in CATdb (http://tools.ips2.u-psud.fr/CATdb)

Table S3. List of 117 genes involved in the relational annotation of *PROSCOOP12* (in addition to Supplementary Fig. S2).

Table S4. Transcriptomic comparison of *proscoop12* and wild-type plants during *E. amylovora* infection.

Supplementary Figures-S1-S7

Supplementary Tables-S1-S4

## Abbreviations

CRP: cysteine-rich peptide
DAMP: damage/danger-associated molecular pattern
PA: phosphatidic acid
PTMP: post-translationally modified peptide
ROS: reactive oxygen species.
