# Supplementary material for: The SCOOP12 peptide regulates defense response and root elongation in Arabidopsis thaliana
Source: J Exp Bot. 2019 Feb 4;70(4):1349–65. doi: 10.1093/jxb/ery454 (PMC6382344; doi:10.1093/jxb/ery454)
Supplement: Supplementary Figures-S1-S7 [file ery454_suppl_supplementary_figures-s1-s7.pdf]

Figure S1

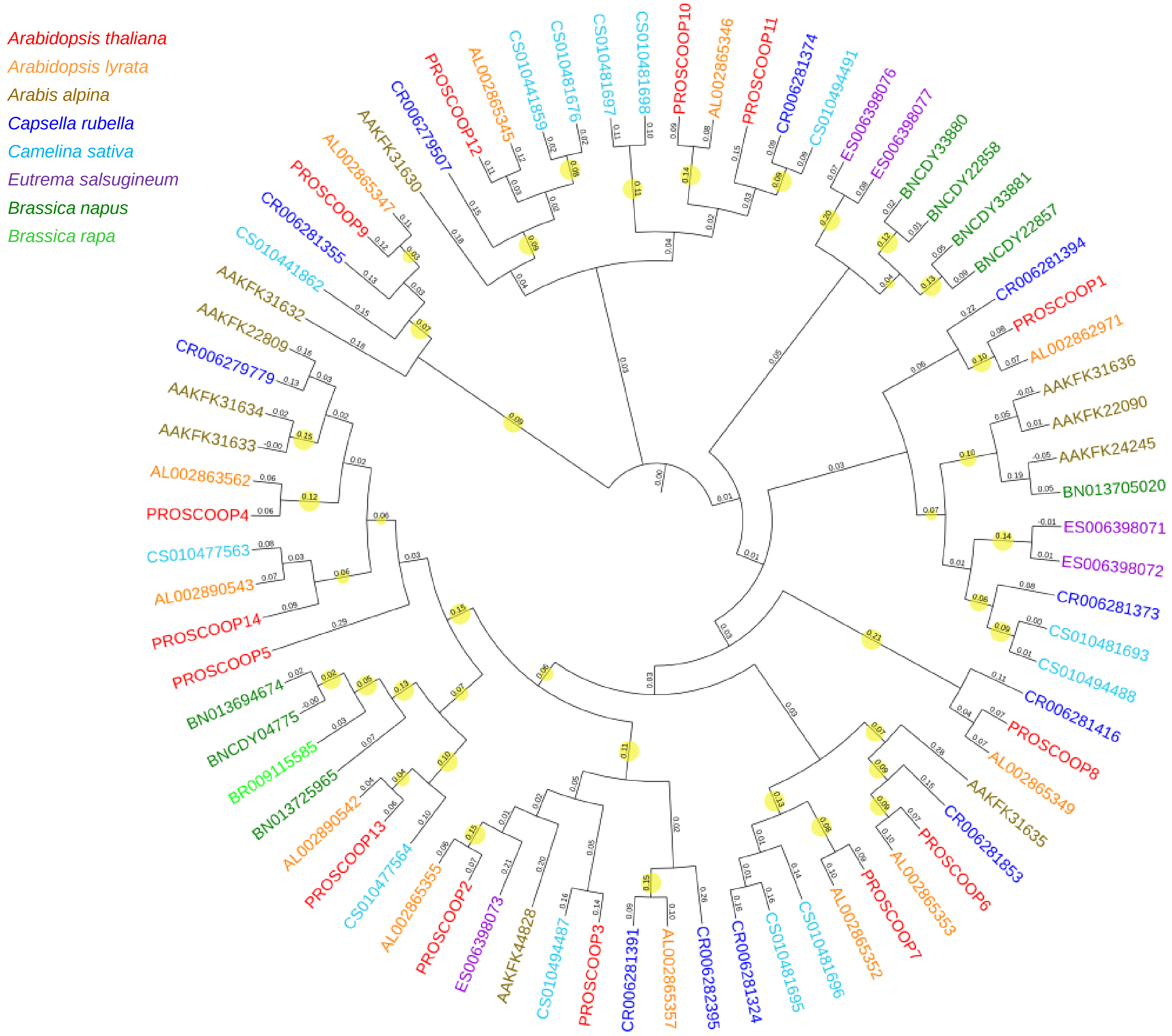

Figure S1. Phylogenetic tree of *PROSCOOP* homologs. The tree was built with the neighbor-joining method from the multiple alignment of 74 homologous Brassicaceae proteins (ClustalX v2.1). Gaps were ignored for tree building and 1000 bootstrap replicates were used to determine the robustness of each node (values higher than 50% are highlighted in yellow). Except for *Arabidopsis thaliana* for which PROSCOOP nomenclature is used, each protein is labelled with two letters (species) and its GenBank ID or XP number.

Figure S2

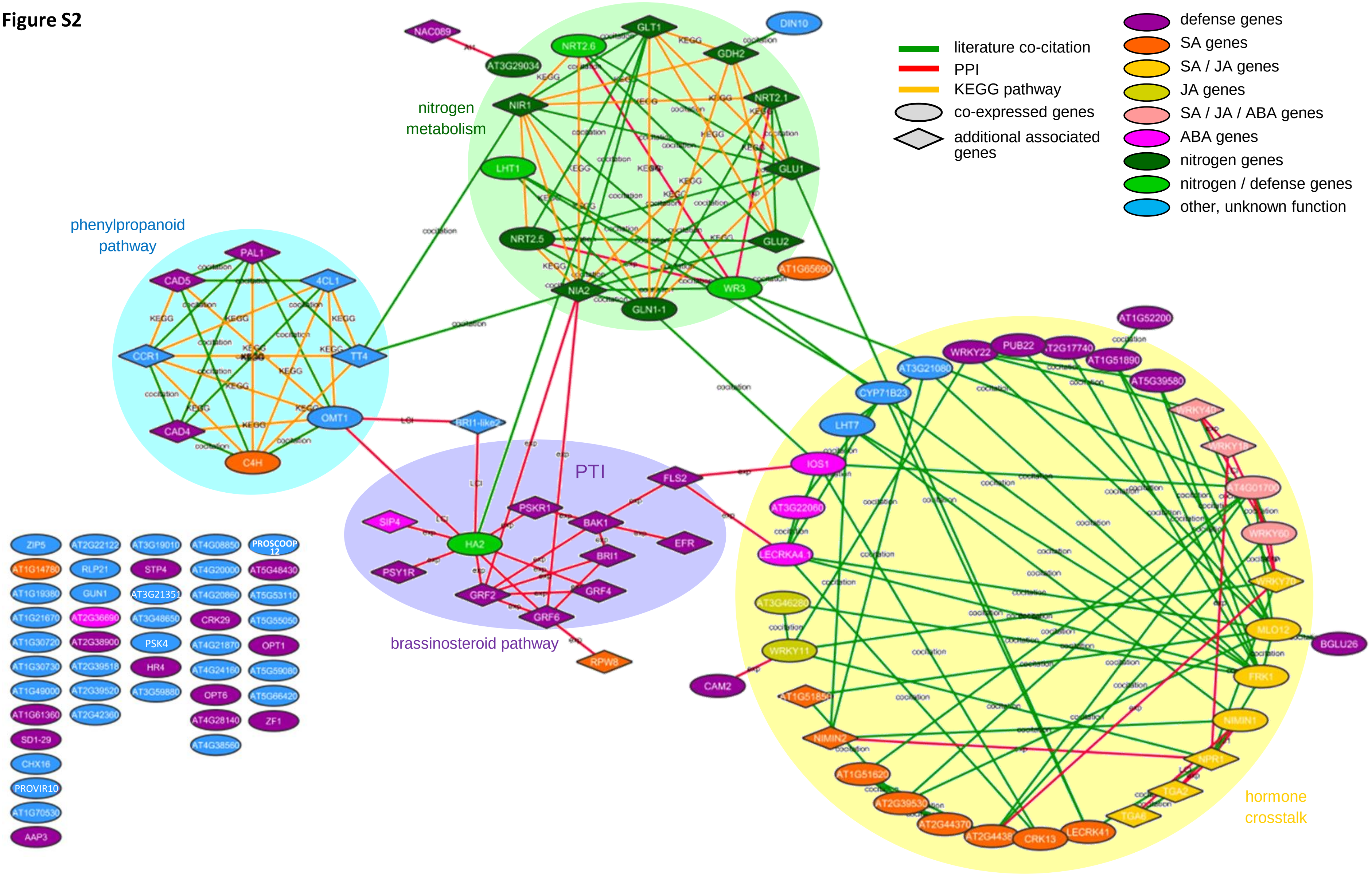

Figure S2. Relational annotation of genes co-expressed with *PROSCOOP12* and their functional partners. Starting from the *PROSCOOP12* gene, the putative partners have been predicted thanks to co-regulation data (GEM2Net resource; Zaag *et al.*, 2015), completed by co-citations in literature/pathway and protein-protein interactions (STRING database, Arabidopsis Interactome and KEGG resources; Szklarczyk *et al.*, 2017). Genes are labelled with their functional names when possible and colored according their biological role, they are all listed with their TAIR names and annotation in the **Table S3A**. Edges are colored according the data explaining the functional links and are detailed in **Table 3B**. The legend is detailed in the top right of the figure.

Figure S3

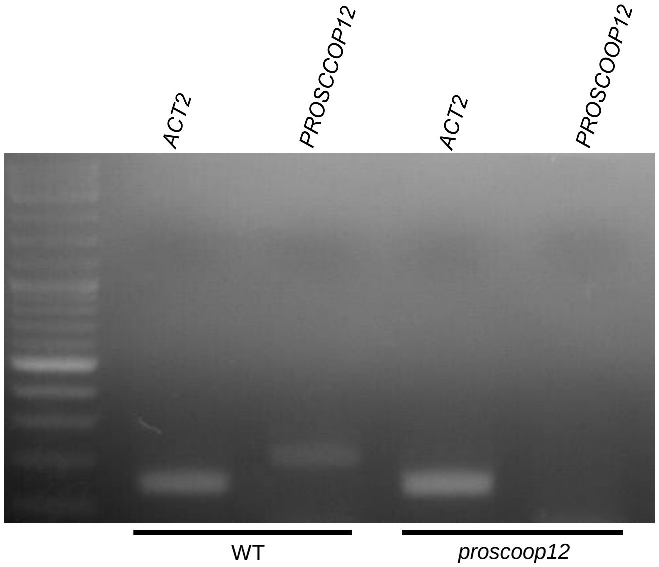

Figure S3. Confirmation of absence of transcription in the *proscoop12* T-DNA knock-out line by RT-PCR. *Actine2* expression (*ACT2*) is used as control.

Figure S4

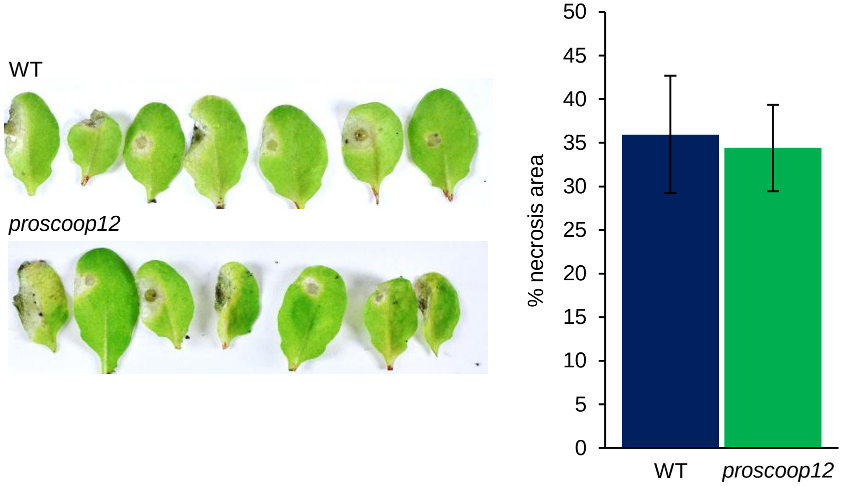

Figure S4. Effect of *A. brassicicola* infection on *proscoop12* leaves. The percentage of necrosis area observed six days after fungal infection is not different between *proscoop12* and wild-type leaves (Student's t-test, error bars show ±SE of the mean).

Figure S5

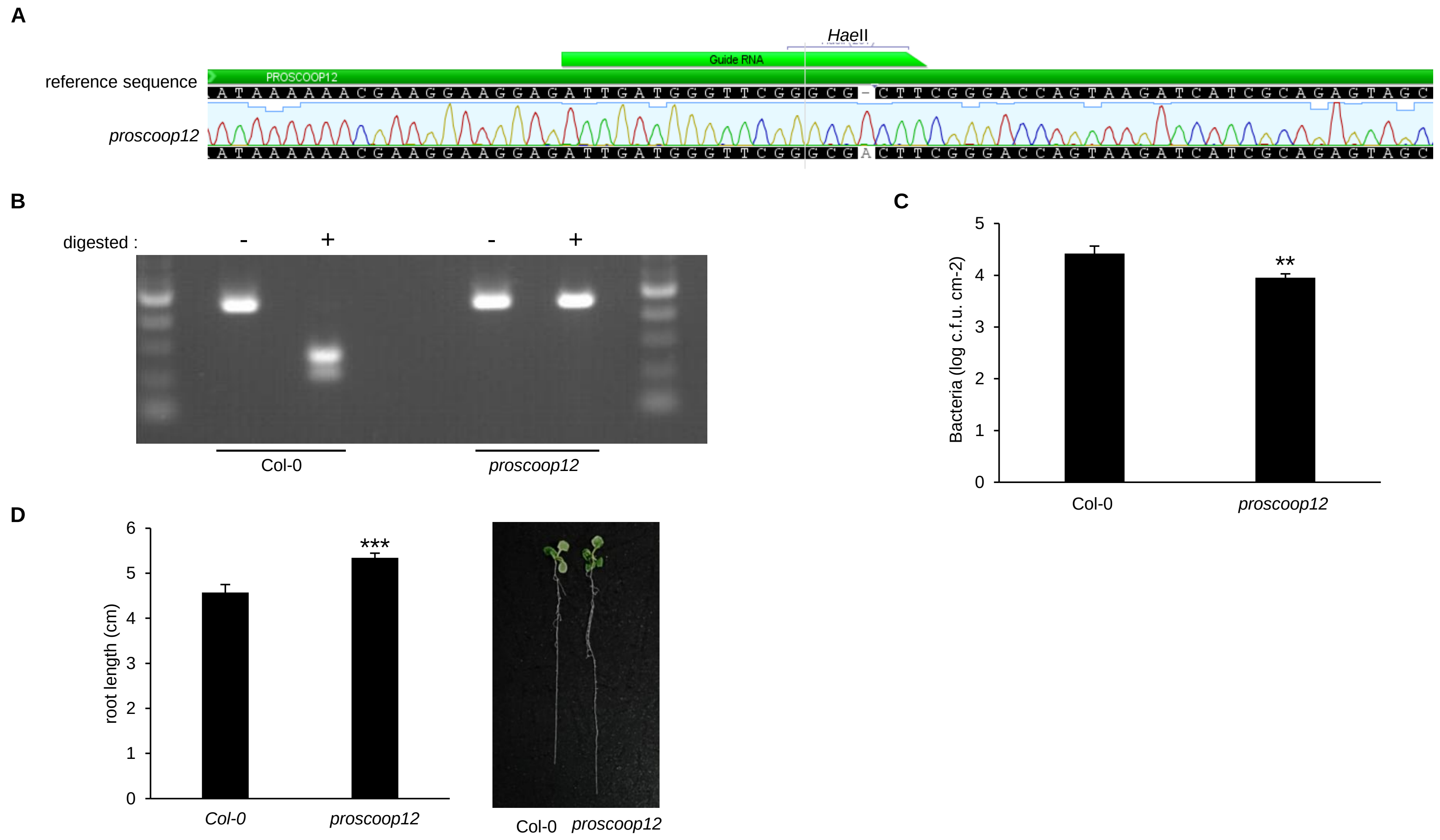

Figure S5. Confirmation of *proscoop12* mutant phenotype in a second genotype. (A) and (B) Mutant genotyping**:** the mutant in Col-0 background was created using the CRISPR-Cas9 approach. The guide-RNA was designed in the first exon. DNA of 15 *proscoop12* mutants was extracted and sequenced. The alignment to the reference sequence shows that the *proscoop12* line has a single base insertion leading to an early stop codon. A 450bp long fragment covering the guide-RNA sequence was amplified by PCR. The PCR product of WT and *proscoop12* DNA was digested using the restriction enzyme *Hae*II. (C) Arabidopsis wild-type (Col-0) plants and CRISPR-Cas9 *proscoop12* mutant were inoculated with *E. amylovora* CFBP1430. Bacteria of 24 leaves were extracted from leaf discs and number of bacteria was quantified after 3dpi. (D) Root growth phenotypes determined after 10 days. Bars represent means of two independent experiments using 25 seedlings. Significant differences according to Student's t-test results: **, P < 0.01; ***, P < 0.001.

Figure S6

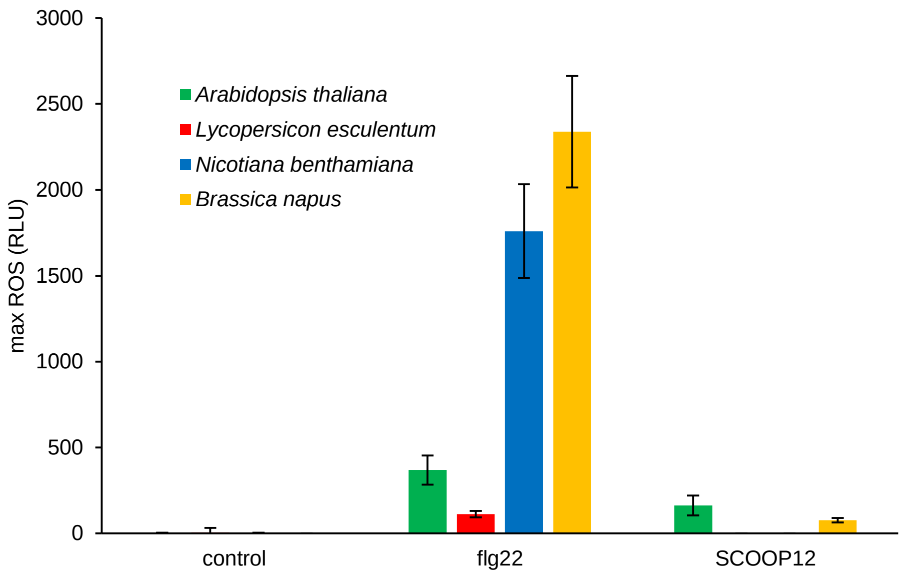

Figure S6. ROS burst measurements on selected plant species treated with SCOOP12. ROS burst assay was performed on *Arabidopsis thaliana* (Col-0), *Lycopersicon esculentum*, *Nicotiana benthamiana* and *Brassica napus*. The flg22 and SCOOP12 peptides were added to a final concentration of 1µM. Bars display the average of the maximum ROS burst in RLU (relative light units) of 12 replicates. Error bars show ±SE of the mean.

Figure S7

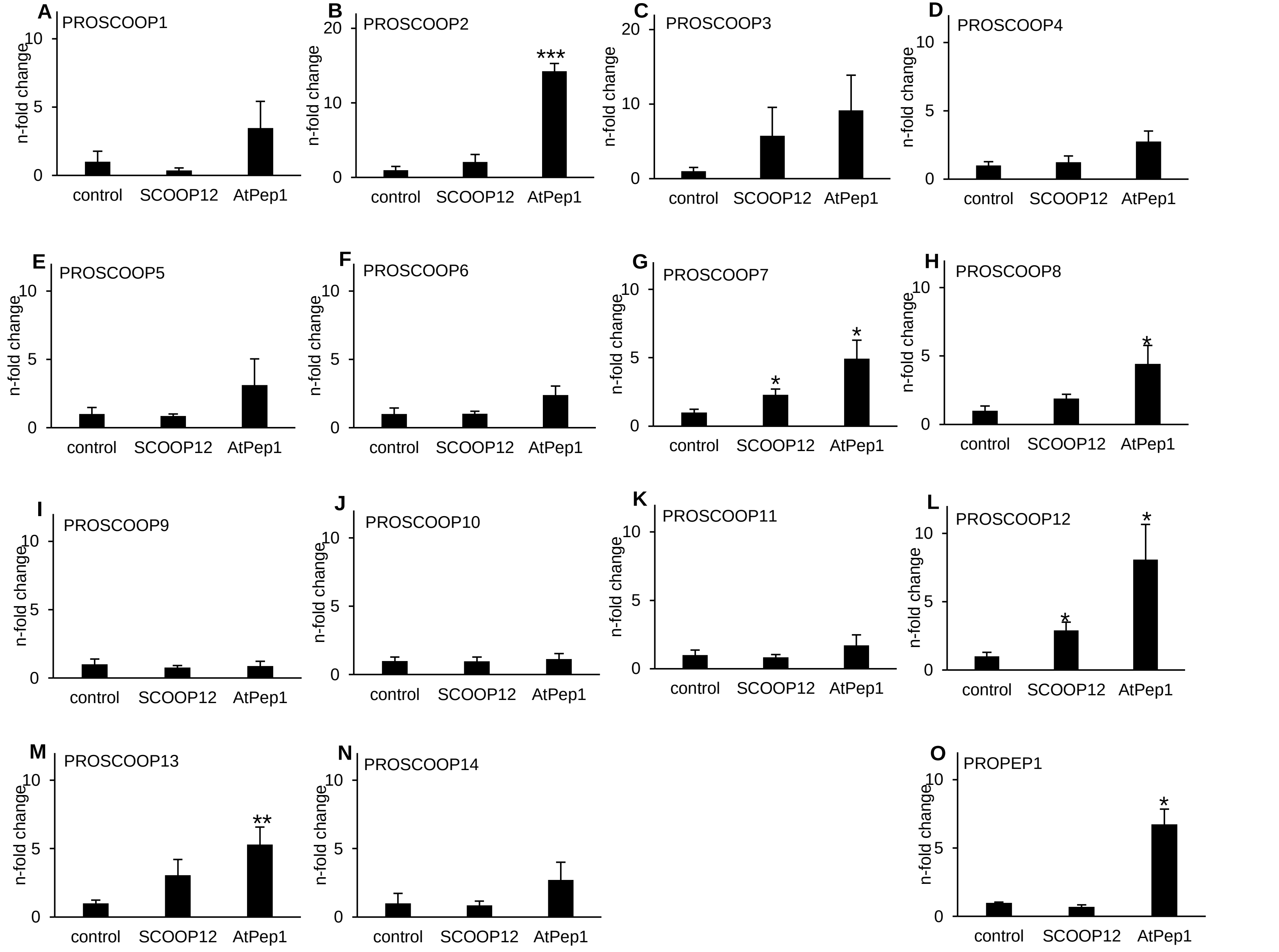

Figure S7. Transcriptional response of the *PROSCOOP* gene family to SCOOP12 and *At*Pep1. SCOOP12 and *At*Pep1 induce the expression of several *PROSCOOP* gene family members. Expression level of the *PROSCOOP* gene family members (A to N) as well as *PROPEP1* (O) were determined by normalization to *ACR12* transcripts, and bars indicate the fold change of transcription relative to the control treatment of at least five independent biological replicates. Error bars show the relative ±SE of the mean. Significant differences according to Student's t-test results: *, $P < 0.05$; **, $P < 0.01$; ***, $P < 0.001$.
